# In Response to: “Using Lean-Based Systems Engineering to Increase Capacity in the Emergency Department

**DOI:** 10.5811/westjem.2014.11.24849

**Published:** 2014-12-15

**Authors:** Benjamin A. White, Yuchiao Chang, Beth G. Grabowski, David F.M. Brown

**Affiliations:** Massachusetts General Hospital, Department of Emergency Medicine, Boston, Massachusetts

In Reply:

We very much appreciate the interest of our colleagues in this important topic, one that has yet to fully mature in the pantheon of emergency medicine literature. We also recognize and noted in our manuscript that the single-site nature of our work is a limitation. However, we disagree that this limitation makes scientific exploration and publication of this nature a fruitless endeavor as implied.

Publication bias aside, the central tenet of Lean methodologies is the elimination of waste in all forms, and thus it is an inherently generalizable tool to improve any process in which waste exists. However, by definition the specific details relating to the underlying process and the implementation of Lean are of key importance, and its potential utility needs to be weighed in each individual setting.

In addition, partly for this reason, we suggest that local processes not affected by the intervention may serve as reliable controls. We specifically used another area of our ED as a control group to avoid the recognized limitations of an analysis that was purely based on data that were obtained “before and after” the changes were implemented.

Finally, Lean methodologies do not always have to be resource intensive as our colleagues suggest. For example, no external consultants or significant internal resources were utilized for this work, and in addition we were in fact able to decrease resource utilization as is common with successful Lean interventions. We respectfully suggest that it is often in this simplicity and focus that Lean interventions, and robust scientific studies describing them, have the potential to be so powerful.

